# A263 IRONING OUT THE DETAILS: CROHN’S DISEASE PATIENT DERIVED MACROPHAGES ARE MORE SUSCEPTIBLE TO FERROPTOSIS

**DOI:** 10.1093/jcag/gwad061.263

**Published:** 2024-02-14

**Authors:** J A Sousa, B E Callejas Pina, Y Munazza, A Wang, M Raman, D McKay

**Affiliations:** University of Calgary, Calgary, AB, Canada; University of Calgary, Calgary, AB, Canada; University of Calgary, Calgary, AB, Canada; University of Calgary, Calgary, AB, Canada; University of Calgary, Calgary, AB, Canada; University of Calgary, Calgary, AB, Canada

## Abstract

**Background:**

Death of intestinal epithelial cells is ubiquitous in Crohn’s disease (CD) but the death of immune cells such as macrophages is less explored. We have found that monocyte derived macrophages from patients with active CD are more susceptible to H_2_O_2_-induced cytotoxicity but the form of regulated cell death these cells undergo remains to be elucidated. Selenium (Se)—a common micronutrient deficiency in patients with CD—is used in the synthesis of selenoproteins that have antioxidant properties (e.g., glutathione peroxidases (GPx)) and are highly expressed in macrophages. Whether Se deficiency plays a role in the increased cytotoxicity also remains to be elucidated.

**Aims:**

To determine the form of cell death of macrophages in response to H_2_O_2_-induced cytotoxicity and whether selenium deficiency plays a role in the increased susceptibility to H_2_O_2_-induced cytotoxicity.

**Methods:**

Blood collected from healthy volunteers and patients with active CD was analyzed for serum GPx activity. Monocytes isolated by plastic adherence were treated with M-CSF (10ng/ml, 7d) to derive macrophages. Macrophages were treated with H_2_O_2_ (500μm, 2h) and lactate dehydrogenase release was measured. *GPX1*, *SLC7A11* and *PTGS2* mRNA expression were determined by qPCR. The presence of cleaved caspase 3 was determined by immunoblotting and IL-1β by ELISA. Lipid peroxidation a marker of ferroptosis was assessed by staining macrophages with Bodipy C-11 (± liproxstatin-1, an inhibitor of ferroptosis).

**Results:**

CD macrophages were two times more susceptible to H_2_O_2_-evoked cell death. In response to H_2_O_2_ macrophages did not express cleaved caspase 3 or IL-1β, but there was an induction of ferroptosis markers (*SLC7A11* and *PTGS2)*. Moreover, lipid peroxidation was induced and could be blocked with liproxstatin-1. Dietary Se intake did not differ between groups but serum GPx activity was greater in patients with CD compared to control. In contrast, *GPX1* mRNA expression and GPx1 protein expression were decreased in CD macrophages compared to healthy controls.

**Conclusions:**

Macrophages derived from patients with CD are inherently more sensitive to ferroptosis potentially through reduced GPx1 expression. Future studies warrant testing if GPx1 prevents ferroptotic macrophage cell death and if it holds therapeutic relevance in CD pathophysiology.

**Funding Agencies:**

Helmsley Charitable Trust, Crohn's and Colitis Foundation of America

